# Salubrinal Ameliorates Inflammation and Neovascularization via the Caspase 3/Enos Signaling in an Alkaline-Induced Rat Corneal Neovascularization Model

**DOI:** 10.3390/medicina59020323

**Published:** 2023-02-09

**Authors:** Gokhan Ozge, Umut Karaca, Mehtap Savran, Gulsah Usta, Kanat Gulle, Murat Sevimli, Fatma Nihan Cankara, Halil Asci

**Affiliations:** 1 Department of Ophthalmology, Gulhane Faculty of Medicine, University of Health Sciences, 06018 Ankara, Turkey; 2 Department of Ophthalmology, Faculty of Medicine, Suleyman Demirel University, 32260 Isparta, Turkey; 3 Department of Pharmacology, Faculty of Medicine, Suleyman Demirel University, 32260 Isparta, Turkey; 4 Department of Histology and Embryology, Faculty of Medicine, Suleyman Demirel University, 32260 Isparta, Turkey

**Keywords:** apoptosis, corneal injury, inflammation, endoplasmic reticulum stress, eNOS

## Abstract

*Background and Objectives:* Ocular alkaline burn is a clinical emergency that can cause permanent vision loss due to limbal stem cell deficiency and corneal neovascularization (CNV). Although the basic pathogenetic mechanisms are considered to be acute oxidative stress and corneal neovascularization triggered by inflammation, the underlying intracellular mechanisms have not been clearly elucidated. The aim of this study was to investigate the role of endoplasmic reticulum (ER) stress on inflammation and neovascularization, and the effect of the ER stress inhibitor salubrinal (SLB), as a novel treatment in a corneal alkaline burn model in rats. *Methods:* Chemical burns were created by cautery for 4 s using a rod coated with 75% silver nitrate and 25% potassium nitrate in the corneal center for the corneal neovascularization (CNV) model. Twenty-eight Wistar albino rats were divided into four groups: SHAM, CNV, CNV + SLB, and CNV + bevacizumab (BVC). After the CNV model was applied to the right eye, a single subconjunctival dose (0.05 mL) of 1 mg/kg salubrinal was injected into both eyes in the CNV + SLB group. A total of 1.25 mg/mL of subconjunctival BVC was administered to the CNV + BVC group. Fourteen days after experimental modeling and drug administration, half of the globes were placed in liquid nitrogen and stored at −20 °C until biochemical analysis. The remaining tissues were collected and fixed in 10% buffered formalin for histopathological and immunohistochemical analysis. Three qualitative agents from three different pathways were chosen: TNFR for inflammation, endothelial nitric oxide synthase (e-NOS) for vascular endothelial growth factor (VEGF)-mediated vascular permeability, and caspase-3 for cellular apoptosis. *Results:* Significantly lower caspase-3 and eNOS levels were detected in the CNV + SLB and CNV + BVC groups than in the CNV group. Additionally, histopathological evaluation revealed a significant decrease in neovascularization, inflammatory cell infiltration, and fibroblast activity in the CNV + SLB and CNV + BVC groups. The endoplasmic reticulum stress inhibitor, salubrinal, administered to the treatment group, attenuated apoptosis (caspase-3) and inflammation (e-NOS). In the control group (left eyes of the SLB group), salubrinal did not have a toxic effect on the healthy corneas. *Conclusion:* The ER stress pathway plays an important role in angiogenesis after alkaline corneal burns, and treatment with SLB modulates this pathway, reducing caspase-3 and eNOS levels. Further studies are needed to understand the molecular mechanisms altered by SLB-mediated therapy. The fact that more than one mechanism plays a role in the pathogenesis of CNV may require the use of more than one molecule in treatment. SLB has the potential to affect multiple steps in CNV pathogenesis, both in terms of reducing ER stress and regulating cellular homeostasis by inhibiting the core event of integrated stress response (ISR). Therefore, it can be used as a new treatment option and as a strengthening agent for existing treatments. Although blockade of intracellular organelle stress pathways has shown promising results in experimental studies, more in-depth research is needed before it can be used in routine practice. To the best of our knowledge, this study is the first to report the role of ER stress in corneal injury.

## 1. Introduction

Ocular alkaline burn is a clinical emergency that requires rapid intervention and can cause permanent corneal damage. Damage to the ocular surface and limbal stem cells can cause permanent visual impairment or blindness [1]. Alkaline chemicals show faster and deeper penetration into the corneal stroma than acidic chemicals do. Although the basic pathogenetic mechanisms are considered to be acute oxidative stress and corneal neovascularization triggered by inflammation, the underlying intracellular mechanisms have not been clearly elucidated [2]. Under cellular stress, increased reactive oxygen species induce inflammation and increase the release of inflammatory cytokines, matrix metalloproteinases, and vascular endothelial growth factor (VEGF) via activation of nuclear factor-kappa B (NF-κB) signaling [3].

**Figure 1:**
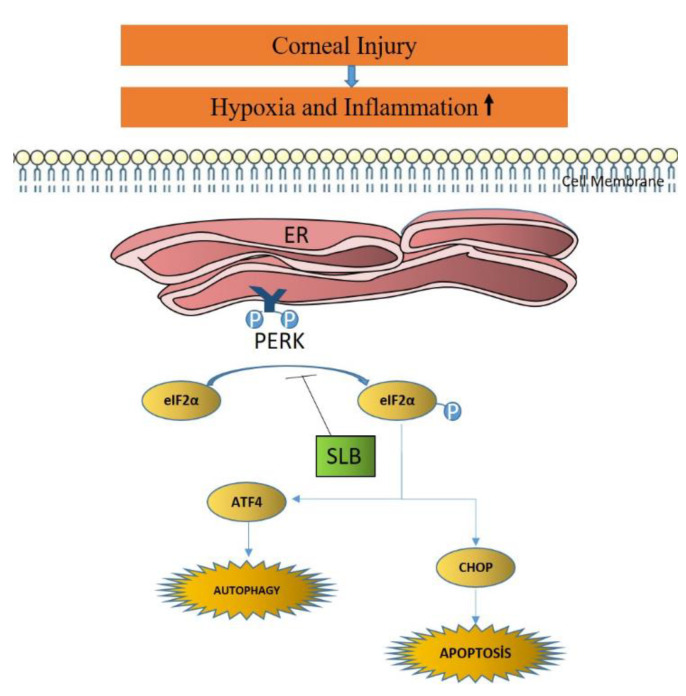
ER stress-mediated apoptosis pathway under corneal injury. Under hypoxic state, PERK involve in apoptosis via eIF2α. The hallmark event is the progressive accumulation of misfolded protein aggregates. Apoptosis is a genetically programmed process aimed at eliminating damaged cells by activation of caspases. The process is activated either by TNFR stimulus extracellularly or intracellularly mainly by nonreceptor stimuli such as DNA damage, ER stress, metabolic stress, UV radiation, or growth factor deprivation. Autophagy, on the other hand, is a cellular catabolic pathway involving protein degradation, organelle cycling, and nonselective degradation of cytoplasmic components.

The endoplasmic reticulum (ER) is an organelle that is responsible for intracellular protein metabolism. After new protein synthesis, folding, and three-dimensional configuration, proteins are transported to intracellular destinations [4]. Inflammatory processes due to infection or trauma cause an increase in unfolded proteins in the ER, which is known as ER stress [5]. Unfolded protein response (UPR) resulting from ER stress results in inhibition of protein synthesis, upregulation of organelles for further protein processing, and initiation of programmed cell death (apoptosis) [6]. However, prolonged ER stress disrupts homeostasis, apoptosis, and the tissue healing process [7]. Three key ER transmembrane proteins, inositol-requiring protein 1 (IRE1), activating transcription factor-6 (ATF6), and protein-kinase-R-like endoplasmic reticulum kinase (PERK), play a major role in UPR-related apoptosis [8]. PERK activation after inflammation and ischemia, ER stress, and accumulation of misfolded and unfolded proteins is mediated by eukaryotic initiation factor 2α (eIF2α) phosphorylation [9]. Salubrinal, a selective eIF2α phosphorylation inhibitor, reduces the load of unfolded proteins by inhibiting eIF2α phosphorylation via PERK inhibition [10] (Figure 1). 

UPR and hypoxia-activated pathways are the two main pathways that aim to create a cytoprotective response by increasing VEGF expression in both physiological (such as placenta) and pathological conditions (tumor vascularization) [11,12]. The interaction between these two pathways has also been studied, and it has been shown that UPR enhances hypoxia-inducible factor α (HIF1 α) activity and VEGF expression [13]. Salubrinal has also been shown to reduce retinal neovascularization by inhibiting the homologous protein (CHOP)-HIF 1 α-VEGF pathways [14]. Although salubrinal is used in experimental studies owing to its anti-inflammatory properties in many tissues, there is no licensed preparation that has been put into clinical use yet. Therefore, salubrinal may be a potential candidate for preventing neovascularization.

Although ER stress-mediated apoptosis that occurs after inflammation has been investigated in the brain, heart, and kidney tissues [8,15,16], its role in the cornea has not been studied. PubMed and Google Scholar were searched between January and May 2019. Unfortunately, limited information was available on ER stress and corneal damage. The aim of this study was to investigate the role of endoplasmic reticulum (ER) stress on inflammation and neovascularization, and the effect of the ER stress inhibitor salubrinal, as a novel treatment in a corneal alkaline burn model in rats.

## 2. Materials and Methods

### 2.1. Animals

All experiments were performed in accordance with the guidelines for animal research from the National Institutes of Health and were approved by the Committee on Animal Research of Suleyman Demirel University, Isparta (No. 28 March 2019-05/03). Twenty-eight female adult Wistar albino rats (250–300 g) were placed in a temperature-(21–22 °C) and humidity-controlled (60 ± 5%) room with a 12:12-h light/dark cycle. All the rats were fed a standard commercial chow diet (Korkuteli Yem, Antalya, Turkey).

The efficacy and safety of a drug candidate molecule should be demonstrated through experimental studies prior to clinical studies. Experimental studies should use the lowest species, phylogenetically. Therefore, rats are a suitable species for corneal studies.

### 2.2. CNV Model and Experimental Design

After intraperitoneal ketamine (90 mg/kg) (Putney Inc., Portland, ME, USA) and xylazine (20 mg/kg) (Vedco Inc., St Joseph, MO, USA) anesthesia, both eyes of the animals were examined and a chemical burn of approximately 2 mm in width was created by cautery for 4 s with a rod coated with 75% silver nitrate and 25% potassium nitrate in the corneal center of the right eye for the corneal neovascularization (CNV) model. This model was described by Mahoney and Waterburry as a practical method for testing anti-inflammatory drugs in the eye [17]. This is one of the most applicable methods among the many models used in the literature.

Rats were divided into four groups (eight in the study group and four in the sham group).

CNV (n = 8): The CNV model was applied to the right eye of the rats, and a single subconjunctival dose (0.05 mL) of dimethyl sulfoxide (DMSO) (276855, Sigma-Aldrich, Merck, Darmstadt, Germany) was injected into the right and left eyes of the rats.

SLB (n = 8): After the CNV model was applied to the right eyes, a single subconjunctival dose (0.05 mL) of 1 mg/kg Salubrinal (SML0951, Sigma-Aldrich, MerckSa, Darmstadt, Germany) was injected into the right and left eyes of the rats. The salubrinal dose was determined in a preliminary study [18].

BVC (n = 8): After the CNV model was applied to the right eyes, a single subconjunctival dose (0.05 mL) of 1.25 mg/mL bevacizumab (Altuzan 100 mg/4 mL, Roche Diagnostics GmbH, Mannheim, Germany) was injected into the right and left eyes of the rats. 

SHAM (n = 4): the CNV model was applied to the right eye. Topical tobramycin (Tobrex; Alcon Labs, Puurs, Belgium) was administered until sacrifice.

Fourteen days after experimental modeling and drug administration, all rats were anesthetized by i.p. injection of 90 mg/kg ketamine (Alfamin, Alfasan, and IBV) and 10 mg/kg xylazine (Alfazin and Alfasan IBV). Half of the globes were placed in liquid nitrogen and stored at −20 °C until biochemical analysis. The remaining tissues were collected and fixed in 10% buffered formalin for histopathological and immunohistochemical analysis.

### 2.3. Hematoxylin Eosin Staining

The eyes were carefully obtained from each animal. Corneal tissues were removed from the globes using surgical techniques and fixed in 10% buffered formaldehyde solution. After fixation, the corneas were washed under running water overnight, dehydrated by passing them through a graded ethanol series, cleared with xylol, and embedded in paraffin. Sections of 4–5 μm thickness were taken from paraffin blocks and stained with hematoxylin and eosin (H&E) for histopathological examination. Light microscopic analysis was performed to evaluate the intensity of neovascularization, inflammatory cell infiltration, and fibroblast activity. The modified form of the scoring system previously defined by Ozdemir et al. was used to evaluate the histopathological findings as follows. Neovascularization: +1, minimal or negative vascularization; +2, limited or focal vascularization; +3, cases intermediate to groups 2 and 4; +4, diffuse and intense vascularization. Intensity of inflammatory cell infiltration: +1, minimal or negative; +2, focal-low infiltration; +3, cases intermediate to groups 2 and 4; +4, intense and diffuse infiltration. Fibroblast activity: +1: minimal or negative activity; +2, focal activity; +3, cases intermediate to groups 2 and 4; +4, diffuse and intense activity [19].

### 2.4. Immunohistochemistry

Caspase-3, e-NOS, and TNFR activity was investigated in corneal sections using immunohistochemical methods. The primary antibodies were caspase-3 (Bioss, Woburn, MA, USA; Cat. No:bs-0081R), e-NOS (Bioss, Cat. No:bs-0163R), TNFR1 (Bioss, Cat. No:bs-2941R). All primary antibodies were diluted with 1:100 Antibody Diluent Reagent Solution (Life Technologies, Carlsbad, CA, USA; Cat. No: 003118). The block solution, streptavidin peroxidase solution, and secondary antibody were used as kits (Cat. No: TP-125-HL). Sections that were 4–5 μm thick were deparaffinized and dehydrated. The sections were then incubated with 3% hydrogen peroxide, Ultra-V Block (ThermoFisher, Waltham, MA, USA), primary antibodies (Bioss), secondary antibody (ThermoFisher), and streptavidin peroxidase (ThermoFisher). Marking with DAB solution (Vectorlab, Burlingame, CA, USA) and nuclear staining with hematoxylin were performed. In this study, a previously reported semiquantitative scoring system was used to evaluate immunoreactivity [20]. Immunostaining intensity was scored as follows: 0, negative; 1, weak; 2, moderate; and 3, strong. The percentages of positively stained cells were calculated and scored as follows: 0–4% = 1, 5–19% = 2, 20–39% = 3, 40–59% = 4, 60–79% = 5, and 80–100% = 6. Finally, multiplicative quick scores were calculated [21].

### 2.5. Statistical Analyzes

GraphPad Prism9 software was used for statistical analysis. One-way analysis of variance (ANOVA) was used to compare the groups, and Tukey multiple comparison test was performed. The *p* < 0.05 value was considered as the level of significance. 

## 3. Results

### 3.1. Histopathology

**Figure 2:**
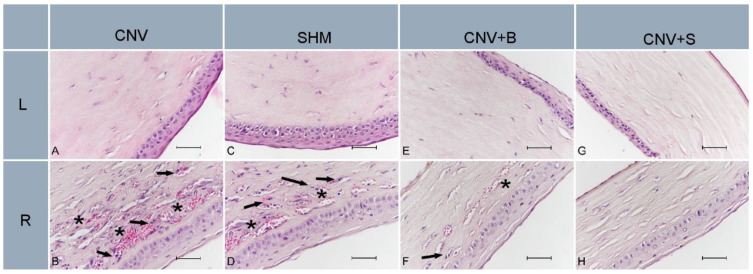
Rat corneal sections. (**A**,**C**,**E**,**G**) Normal corneal histology in CNV-L, SHM-L, CNV + B-L, and CNV + S-L groups, respectively. (**B**,**D**) Extensive and intense neovascularization, significant inflammatory cell infiltrations, and increased fibroblast activity in the subepithelial area in CNV-R and SHM-R groups. (**F**,**H**) A significant decrease in histopathological findings in CNV + B-R and CNV + S-R groups. (**H**,**E**) Staining, ×40, scale bar = 50 µm (arrows show inflammatory cell infiltrations, asterisk shows neovascularization areas).

In all groups, no histopathological findings were observed in the left cornea where the corneal neovascularization model was not applied (Figure 2A,C,E,G). Extensive neovascularization, significant inflammatory cell infiltration, and increased fibroblast activity were observed in the subepithelial area of the CNV-R (right eye) (Figure 2B) and SHAM-R (Figure 2D) groups. There were no statistically significant differences between the two groups in any of the histopathological findings (all *p* > 0.05). Significant decreases in neovascularization, inflammatory cell infiltration, and fibroblast activity were detected in the BVC-R (Figure 2F) group sections compared with the CNV-R (*p* < 0.001, *p* < 0.01, and *p* < 0.01, respectively) and SHAM-R (*p* < 0.01, *p* < 0.01, and *p* < 0.05, respectively) groups. Similar to the BVC-R group, in the SLB-R (Figure 2H) group sections, a significant decrease in neovascularization, inflammatory cell infiltration, and fibroblast activity was detected compared to the CNV-R (*p* < 0.001, *p* < 0.01, and *p* < 0.01, respectively) and SHAM-R (*p* < 0.001, *p* < 0.01, and *p* < 0.01, respectively) groups. There was no statistically significant difference in the intensity of histopathological findings between the BVC-R and SLB-R groups (all *p* > 0.05). The findings are summarized in Table 1.

### 3.2. Immunohistochemistry

**Figure 3:**
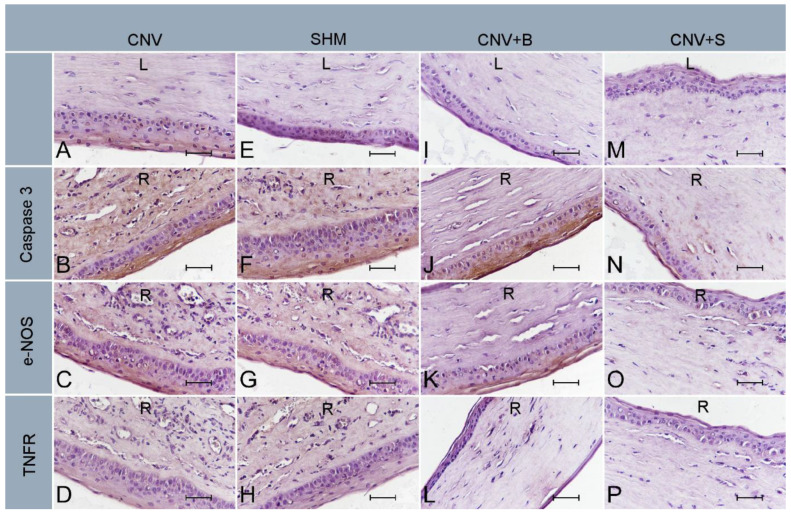
Immunohistochemical stainings. No immunoreactivity was observed in CNV-L (**A**), SHM-L (**E**), CNV + B-L (**I**), or CNV + S-L (**M**) groups with caspase-3, e-NOS, and TNFR. Increased caspase-3 immunoreactivity in CNV-R (**B**) and SHM-R (**F**) groups and negative capase-3 immunoreactivity in CNV + B-R (**J**) and CNV + S-R (**N**) groups. A slight increase in e-NOS immunoreactivity in CNV-R (**C**) and SHM-R (**G**) groups and negative e-NOS immunoreactivity in CNV + B-R (**K**) and CNV + S-R (**O**) groups. Negative immunoreactivity with TNFR in CNV-R (**D**), SHM-r (**H**), CNV + B-R (**L**), and CNV + S-R (**P**) groups. ×40, scale bar = 50 µm.

Table 2 presents the semiquantitative scoring results for caspase-3, endothelial nitric oxide synthase (e-NOS), and tumor necrosis factor receptor (TNFR) immunoreactivity in all the groups. According to these results, with caspase-3, e-NOS, and TNFR, no immunoreactivity was observed in the CNV-L (left eye) (Figure 3A), SHAM-L (Figure 3E), BVC-L (Figure 3I), and SLB-L (Figure 3M) groups. Compared to these groups, caspase-3 and e-NOS activities were significantly increased in the CNV-R (Figure 3B,C) and SHAM-R (Figure 3F,G) groups (all *p* < 0.001). Compared to the CNV-R and SHAM-R groups, a significant decrease in caspase-3 and e-NOS immunoreactivity was observed in the BVC-R (Figure 3J,K) and SLB-R (Figure 3N,O) groups (all *p* < 0.001). TNFR immunoreactivity was negative in all groups (Figure 3D,H,L,P). 

## 4. Discussion

In a preliminary study, we aimed to determine whether endoplasmic reticulum stress inhibition affects inflammation and neovascularization after alkaline burn and to determine the effective dose of salubrinal [18]. In this study, it was observed that inflammation and neovascularization were triggered in an alkali burn model created in experimental animals (CNV group). It is obvious that in post-traumatic restoration of the cornea, which should be avascular, neovascularization results in permanent vision loss. The endoplasmic reticulum stress inhibitor salubrinal, administered to the treatment group, attenuated apoptosis (caspase-3) and inflammation (e-NOS). In the control group (left eyes of the SLB group), salubrinal did not have a toxic effect on the healthy corneas.

Oxidative stress and inflammation are closely related pathological mechanisms that occur following injury. Antioxidant and anti-inflammatory systems, which are rapidly activated in healthy tissues, aim to heal with minimal tissue damage [22]. Recently, a number of pathways that indicate stress in intracellular organelles after inflammation have been identified. ER stress develops secondary to inflammation, especially in cerebral and metabolic diseases, and causes permanent damage if prolonged [8,10,23]. 

In recent years, the endoplasmic reticulum (ER) stress pathway has been investigated as an intracellular therapeutic pathway triggered by inflammation in many tissues. Although postdamage ER stress is desirable in healthy cells, prolonged activation can induce damage mechanisms, such as apoptosis and autophagy [24]. Activation of the ER stress pathway may contribute to angiogenesis and inflammation in retinal vascular endothelial cells in a high-dose glucose-induced diabetic retinopathy model [25]. Similarly, Wang et al. demonstrated the ER stress pathway in the retinal neovascular process as a novel target mechanism for anti-inflammatory and antivascular therapy [26]. To the best of our knowledge, this is the first study to investigate the ER stress pathway in a corneal alkaline-burn model. 

Proapoptotic mechanisms that develop after ER stress may be mediated either by receptors (PERK, ILE-1, and ATF4-) or mitochondria. Caspase-3 levels were affected in both pathways. Impaired ER function after ischemia has been shown to be ameliorated by eIF2a phosphorylation and UPR suppression [27]. In our study, we showed that after inhibition of eIF2a phosphorylation with salubrinal, neovascularization, inflammatory cell migration, and fibroblast activity regressed. Simultaneously, salubrinal can protect corneal cells from apoptosis, as evidenced by caspase-3 inactivation immunohistochemically. Moreover, histopathological and immunohistochemical data were similar for both BVC and SLB. Our data strongly support the idea that SLB exerts similar effects to BVC, albeit through different mechanisms, suggesting that SLB could be used for the treatment of inflammation-induced neovascularization. 

The overall cellular response to the stress stimulus is the integrated stress response (ISR), which involves ER stress and aims to maintain cellular hemostasis. ISR is triggered by many factors such as viral infection or hypoxia. ISR plays an important role in cellular hemostasis, and the core event involved in this process is eIF2a phosphorylation [28]. In the physiological cellular cycle, eIF2α phosphorylation aids cell survival and recovery by allowing the translation of selected genes through the activation of transcription factor 4 (ATF4). However, severe or prolonged cellular stress triggers the accumulation of unfolded proteins, and this process evolves into the autophagy/apoptosis pathway. SLB may inhibit ISR-regulated cellular damage by inhibiting eIF2a phosphorylation [29,30]. 

The toxicity of salubrinal, as a novel treatment agent, is another issue. Since it is a new agent, no in vitro or in vivo toxicity studies have been conducted and published. The only published toxicity study has been conducted in silico. In this study, a comprehensive in silico toxicity assessment of salubrinal and its analogs was performed, and it was shown that almost all of the 55 structures examined were not toxic to rats [31]. However, the study also predicted the possible cardiotoxic, hepatotoxic, and immunotoxic effects of salubrinal, and the researchers were especially aware of heart and liver studies. These results must be supported by further studies, because, on the contrary, salubrinal has been shown to treat cellular death due to various chemical or biological toxic agents in the literature [32,33]. In this study, we did not observe any toxic side effects in the healthy corneas. In addition, no toxic effects have been reported in eye studies. 

The avascular form and physiology of the cornea predispose it to oxidative damage and cell loss (apoptosis). In addition to the above-mentioned mechanisms (ER stress), mitochondrial stress has also been shown to contribute to the pathogenesis of corneal diseases [34]. Mitophagy is a specific type of autophagy that involves the breakdown of damaged mitochondria, which accumulates due to prolonged stress and regulates mitochondrial regulation. Mitophagy can be observed in many diseases in response to mitochondrial DNA damage caused by a variety of intracellular or extracellular stresses. Oxidative stress and mitochondrial dysfunction have been shown to worsen and accelerate the course of diseases such as Fuchs endothelial corneal dystrophy, Kearns–Sayre syndrome, and keratoconus [35]. Mitochondrial oxidative stress blockade together with ER stress blockade (salubrinal) may have a synergistic effect and can be used as a new treatment target.

In recent years, many studies have investigated the role of vascular endothelial growth factor (VEGF) in corneal damage and subsequent CNV. VEGF inhibition has been proven to be a powerful treatment for CNV [36]. Bevacizumab (Avastin; Roche, Welyn Garden City, UK) is a recombinant humanized anti-VEGFA monoclonal antibody. Bevacizumab has been proven to exert a therapeutic effect on neovascularization after topical, subconjunctival, perilimbal, or intrastromal applications [37]. Therefore, in our study, we compared the efficacy of salubrinal and bevacizumab (although these are not approved treatment protocols for corneal neovascularization).

The most important limitation of this study is that biomarkers of inflammation and apoptosis were not analyzed by more detailed methods such as Western blot analysis or polymerase chain reaction. The effects of salubrinal on VEGF expression were also evaluated. Additionally, the lack of quantitative measurements of corneal epithelialization using photography is another limitation.

In this study, we aimed to investigate the role of endoplasmic reticulum (ER) stress in inflammation and neovascularization and the usability of salubrinal as a new therapeutic agent. Therefore, three qualitative agents from three different pathways were chosen: TNFR-for inflammation [38], e-NOS- for VEGF-mediated vascular permeability [39], and caspase-3 for cellular apoptosis [40]. The final study (currently in the draft phase) aimed to present the amendatory effect of salubrinal on all overactivated pathways of inflammation with quantitative agents. Hence, we examined EIF2-α phosphorylation by Western blot; CHOP, PERK, and caspase 12 expression by PCR; as well as VEGFR2, eNOS (permeability), and COX2 (PGI2 production-vasodilation) expression.

## 5. Conclusions

The ER stress pathway plays an important role in angiogenesis after alkaline corneal burns, and treatment with SLB modulates this pathway, reducing caspase-3 and eNOS levels. Further studies are needed to understand the molecular mechanisms altered by SLB-mediated therapy. The fact that more than one mechanism plays a role in the pathogenesis of CNV may require the use of more than one molecule in treatment. SLB has the potential to affect multiple steps in CNV pathogenesis, both in terms of reducing ER stress and regulating cellular homeostasis by inhibiting the core event of ISR. Therefore, it can be used as a new treatment option and as a strengthening agent for existing treatments. Although blockade of intracellular organelle stress pathways has shown promising results in experimental studies, more in-depth research is needed before it can be used in routine practice.

## Figures and Tables

**Table 1:** The mean (±SD) results for histopathological findings in all groups. (Compared to CNV-R; ^#^: *p* < 0.05, **: *p* < 0.01, ***: *p* < 0.001).

	CNV-L	CNV-R	SHAM-L	SHAM-R	BVC-L	BVC-R	SLB-L	SLB-R
**Neovascularization**	1.2 ± 0.4	3.8 ± 0.4	1.2 ± 0.4	3.6 ± 0.5 ^#^	1.2 ± 0.4	2.2 ± 0.4 ***	1.2 ± 0.4	2.0 ± 0.7 ***
**Inflammatory Cell Infiltration**	1.2 ± 0.4	3.6 ± 0.5	1.2 ± 0.4	3.4 ± 0.5 ^#^	1.2 ± 0.4	2.2 ± 0.4 **	1.2 ± 0.4	2.2 ± 0.4 **
**Fibroblast Activity**	1.2 ± 0.4	2.8 ± 0.4	1.2 ± 0.4	2.6 ± 0.5 ^#^	1.2 ± 0.4	1.6 ± 0.5 **	1.2 ± 0.4	1.4 ± 0.5 **

CNV, corneal neovascularization. BVC, bevacizumab SLB: salubrinal R: right eye; L: left eye.

**Table 2:** Multiplicative quick score results of caspase-3, e-NOS, and TNFR immunohistochemistry. Data are indicated as median (Q25–Q75), minimum, and maximum values. (Compared to L eyes *: *p* < 0.001, compared to CNV-R: ^#^: *p* >0.05, **: *p* < 0.001).

		CNV-L	CNV-R	SHAM-L	SHAM-R	BVC-L	BVC-R	SLB-L	SLB-R
**CASPASE-3**	Med. (Q25-Q75)	0(0–1)	9(9–12) *	0(0–0.5)	9(6–10.5) *^,#^	0(0–1)	1(0–1) **	0(0–0)	0(0–0.5) **
	Min.	0	9	0	6	0	0	0	0
	Max	1	12	1	12	1	0	0	1
**e-NOS**	Med. (Q25-Q75)	0(0–0.5)	6(4–7.5) *	0(0–0)	6(4–7.5) *^,#^	0(0–0.5)	0(0–1) **	0(0–0)	0(0–0.5) **
	Min.	0	4	0	4	0	0	0	0
	Max	1	9	0	9	1	1	0	1
**TNFR**	Med. (Q25-Q75)	0(0–0)	0(0–1)	0(0–0)	0(0–1)	0(0–0)	0(0–0)	0(0–0)	0(0–0)
	Min.	0	0	0	0	0	0	0	0
	Max	0	1	0	1	0	0	0	0

CNV, corneal neovascularization. BVC, bevacizumab SLB: salubrinal R: right eye; L: left eye.

## Data Availability

The datasets used and/or analyzed during the current study are available from the corresponding author upon reasonable request. Unfortunately, these data are not publicly available because of local data protection laws.
